# High-Resolution Microbiome Profiling for Detection and Tracking of *Salmonella enterica*

**DOI:** 10.3389/fmicb.2017.01587

**Published:** 2017-08-18

**Authors:** Christopher J. Grim, Ninalynn Daquigan, Tina S. Lusk Pfefer, Andrea R. Ottesen, James R. White, Karen G. Jarvis

**Affiliations:** ^1^Center for Food Safety and Applied Nutrition, United States Food and Drug Administration, Laurel MD, United States; ^2^Center for Food Safety and Applied Nutrition, United States Food and Drug Administration, College Park MD, United States; ^3^Resphera Biosciences, Baltimore MD, United States

**Keywords:** 16S rRNA, community, profiling, microbiome, *Salmonella*, metagenomics

## Abstract

16S rRNA community profiling continues to be a useful tool to study microbiome composition and dynamics, in part due to advances in next generation sequencing technology that translate into reductions in cost. Reliable taxonomic identification to the species-level, however, remains difficult, especially for short-read sequencing platforms, due to incomplete coverage of the 16S rRNA gene. This is especially true for *Salmonella enterica*, which is often found as a low abundant member of the microbial community, and is often found in combination with several other closely related enteric species. Here, we report on the evaluation and application of Resphera Insight, an ultra-high resolution taxonomic assignment algorithm for 16S rRNA sequences to the species level. The analytical pipeline achieved 99.7% sensitivity to correctly identify *S. enterica* from WGS datasets extracted from the FDA GenomeTrakr Bioproject, while demonstrating 99.9% specificity over other *Enterobacteriaceae* members. From low-diversity and low-complexity samples, namely ice cream, the algorithm achieved 100% specificity and sensitivity for *Salmonella* detection. As demonstrated using cilantro and chili powder, for highly complex and diverse samples, especially those that contain closely related species, the detection threshold will likely have to be adjusted higher to account for misidentifications. We also demonstrate the utility of this approach to detect *Salmonella* in the clinical setting, in this case, bloodborne infections.

## Introduction

*Salmonella* infection is a common bacterial disease throughout the developed and developing world, causing considerable morbidity and mortality. Salmonellosis can be categorized into two disease manifestations, a more common, and usually self-limiting, gastroenteritis due to non-typhoidal salmonellae (NTS) and the rarer, more severe, typhoid fever. Worldwide, it is estimated that NTS cause 94 million cases of gastroenteritis and 155,000 deaths per year (Majowicz et al., 2010), and in the United States, NTS are estimated to cause one million cases, 19,000 hospitalizations, and 380 deaths, annually (Dvir et al., 2014). The majority of non-typhoidal *Salmonella* cases are due to the consumption of contaminated food and water, and as such, this group of organisms is considered a major foodborne pathogen, occurring in almost all types of foods. Additionally, transmission of the organism can occur due to the handling of contaminated farm animals or reptiles.

It is of paramount importance to rapidly and accurately identify foods that are contaminated with foodborne pathogens, such as *Salmonella*, as well as possible sources of contamination, to minimize public health burden. There are numerous culture-based detection and isolation protocols to detect foodborne pathogens, many of which are dependent on pathogen-commodity pairings, such as those in the Food and Drug Administration (FDA) Bacteriological Analytical Manual (BAM) and the United States Department of Agriculture (USDA) Microbiological Laboratory Guidebook (MLG). These methods rely on an array of selective and differential culture media to isolate colonies of the target pathogen, which are used for further characterization, such as sub-typing, serotyping, antibiotic susceptibility testing, and whole genome sequencing (WGS) (Humphries and Linscott, 2015). For example, detection of *Salmonella* generally involves an overnight non-selective enrichment, followed by an overnight selective enrichment in Rappaport Vassiliadis (RV) and Tetrathionate (TT) broths, followed by plating on selective media, such as Xylose lysine deoxycholate (XLD) or Xylose-Lysine-Tergitol 4 (XLT4) agar plates (Andrews et al., 2016). Suspect colonies are confirmed and then further characterized.

Although culture methods are the gold standard in microbiology due to their high sensitivity, current and emerging molecular methods, most notably, PCR, have greatly improved the time to detection for target microorganisms. Mass spectrometry-based methods, such as MALDI-TOF, have also shown great promise in detection and identification of bacterial pathogens. The use of WGS and the application of metagenomics have rapidly increased due to technological advances and cost-reduction in high-throughput next-generation sequencing (Kuczynski et al., 2011a). This technology has in turn become more accessible, and its application allows the characterization of an entire microbial community, in addition to multiple target pathogens (Jarvis et al., 2015; Ottesen et al., 2016).

Utilizing shotgun metagenomics and 16S rRNA gene sequencing is advantageous for microbiome profiling as well as targeted detection assays, because it is culture-independent and relatively unbiased compared to traditional culture methods that rely on highly selective media. While shotgun metagenomics can provide a better understanding of dominant microbial community members, such as identification to the species level, sub-typing and resistome characterization, this method is more expensive due to the requirement of deeper sequencing for higher genome coverage. Microbiome profiling through 16S rRNA gene sequencing provides an economical approach to analyze microbial communities’ dynamics, from a taxonomic perspective; tracking and identifying changes in that population structure in response to experimental variables. While this method is reliable for higher taxonomic levels, species-level inference with 16S rRNA gene sequencing has remained a significant challenge, particularly for accurate identification of *Salmonella enterica* and other enteric pathogens. Previously, we evaluated the performance of Resphera Insight, a high-resolution 16S rRNA sequence analysis pipeline, for identification of *Listeria monocytogenes* from naturally contaminated ice cream samples (Ottesen et al., 2016). We also recently applied this tool for tracking of *S. enterica* in a modified US FDA BAM protocol for detection of *Salmonella* through reduced enrichment (Daquigan et al., 2016). In this study, we performed an expanded evaluation of this algorithm for detection of *S. enterica* and evaluated factors affecting the identification of this pathogen in food and clinical samples.

## Materials and Methods

### Diagnostic True Positive Rate for *S. enterica* Detection

To perform an evaluation of the species-level accuracy for *S. enterica*, 512 randomly selected *S. enterica* whole-genome shotgun sequencing datasets from the FDA GenomeTrakr Project (NCBI Project ID PRJNA183844) were obtained from the NCBI Sequence Read Archive (Supplementary Table S1). Paired-end MiSeq fastq sequences were first filtered based on quality (Q20) and length (150 bp), and then overlapping sequences were merged using FLASH (Magoc and Salzberg, 2011). Merged reads and any unmerged Read 1 sequences were then screened for 16S rRNA fragments using Bowtie2 (Langmead and Salzberg, 2012) against a broad database of 16S rRNA sequences. An additional layer of BLAST-based filtering was used to confirm location-specific query matches to the *S. enterica* subsp. *enterica* serovar Typhimurium strain LT2 16S rRNA gene (NCBI accession NR_074910.1). Valid, filtered sequences were submitted to Resphera Insight (Baltimore, MD, United States^1^) for high-resolution taxonomic identification. Briefly, the tool relies on a manually curated 16S rRNA database of 11,000 species with a re-structured bacterial taxonomy and a hybrid global-local alignment strategy to assign sequences a high-resolution taxonomic lineage. The approach attempts to achieve species-level resolution when possible, but when the underlying statistical model indicates uncertainty in final species membership, the tool minimizes false positives by providing “ambiguous assignments” i.e., a list of species reflecting the uncertainty. For example, if a 16S fragment is ambiguous between *S. enterica* and *S. bongori*, the algorithm will provide the assignment: “*Salmonella*_*bongori*:*Salmonella*_*enterica.*”

Here, our statistic of performance sensitivity is the Diagnostic True Positive rate (DTP), defined as the percentage of 16S rRNA sequences that were correctly and unambiguously assigned to *S. enterica.* For comparison, we evaluated diagnostic performance for the RDP classifier and UCLUST-REF algorithms implemented within QIIME (Kuczynski et al., 2012; Navas-Molina et al., 2013), using default parameter settings. We also sought to apply the standard taxonomic characterization tools utilized by the MOTHUR (v1.35) and DADA2 (v1.0) packages (Schloss et al., 2009; Callahan et al., 2016); however, by default, these tools do not make species-level assignments. Therefore, they were excluded. The expanded validation of the Insight method was then performed on all available *S. enterica* isolates annotated in GenomeTrakr at the time of the study (*n* = 12,090, Supplementary Table S2). Of this expanded validation dataset, 93 SRA datasets from GenomeTrakr were determined to be mis-annotated as *Salmonella* or reflected mixtures of microbial species based on WGS analysis using MetaPhlAn (Data not shown). To provide an unbiased evaluation of all three algorithms, four different size classes of sequencing read lengths were evaluated for the effect of this parameter on DTP. Further, DTP rates were determined for each position of the 16S rRNA gene, from nucleotide positions 25 to 1,000. This approach was used to account for difference in 16S rRNA amplification and sequencing strategies.

### Evaluation of *S. enterica* False Positive Rate

An evaluation of the false positive rate of Resphera Insight to accurately detect *S. enterica* was performed using 16S rRNA amplicon datasets reflecting raw milk cheese samples spiked with variable levels of *Escherichia coli* (O157:H7 or O103). *In silico* simulations for related *Enterobacteriaceae* were performed by first aligning primer sequences (V1–V3 or V3–V4 region) to corresponding reference 16S rRNA genes to determine exact coordinates of the expected amplicon sequences, and subsequently generating sequences from those coordinates using a random nucleotide error rate of 0.5% (10,000 sequences per species for both regions).

### Bacterial Strains for Inoculation

Cilantro was spiked with *Salmonella* Newport, *S*. Tennessee, or *S*. Thompson. Ice cream samples were spiked with *S.* Enteritidis or *S*. Typhimurium. Chili powder samples were spiked with *S.* Montevideo. Frozen stocks of *S. enterica* were streaked onto Trypticase Soy Agar plates (Difco^TM^, Sparks, MD, United States) and incubated at 35°C overnight. Bacterial cell suspensions were prepared in 0.85% sterile saline to approximately 0.5 McFarland turbidity units, equivalent to 1 × 10^8^ CFU/mL, and serially diluted to approximately 28 CFU/mL for inoculation. Biosafety Level 2 practices, containment equipment, and facilities were employed for all experiments involving Biosafety Level 2 pathogens. All sample manipulations were performed within a certified Class II biological safety cabinet (BSC). Standard personnel protective equipment (PPE) was used, such as disposable gloves, laboratory coats, and eye protection.

### Preparation of Inoculated Food Matrices

Cilantro was purchased from a local grocery store or provided by the Department of Agriculture and Rural Development in Lansing, Michigan, and then stored at 4°C until used. Ice cream, naturally contaminated with *L. monocytogenes*, was stored at -20°C until used. Chili powder samples were supplied by the FDA Northeast Regional Laboratory. To prepare samples, 25 g of cilantro or chili powder, and 150 g of ice cream were aseptically portioned into sterile Whirlpak bags (Nasco; Fort Atkinson, WI, United States) and inoculated with *Salmonella* at a ratio of 1–2 CFU per gram for cilantro and ice cream and at four levels for chili powder, ultra-low (0.5 CFU/gm), low (5 CFU/gm), medium (51 CFU/gm), and high (512 CFU/gm). Cilantro samples were also “aged” at 4°C for 48–72 h to simulate natural contamination.

### *Salmonella* Enrichment from Food

Following the FDA BAM method for *Salmonella* detection (Andrews et al., 2016), chili powder and aged cilantro samples were pre-enriched in a non-selective broth for 24 h (24-h samples), and then aliquots were transferred to selective RV and TT broths for a secondary 24 h enrichment (48-h samples). Ice cream samples were enriched for 48 h following the FDA BAM method for *Listeria* but without acriflavin, cycloheximide, and nalidixic acid (Hitchins et al., 2016). For all foods, a 10 μL aliquot was streaked onto XLT4 (Becton, Dickinson and Company, Sparks, MD, United States) agar plates at two time points, 24 and 48 h, and plates were incubated for 24 h at 35°C. Suspect black colonies observed on XLT4 agar plates were confirmed as *Salmonella* using the VITEK 2 system (BioMérieux, France). Only samples with confirmed *Salmonella* were considered culture-positive. Microbiome cell pellets were also collected at each 0, 24, and 48-h time point, centrifuged at 7,100 × *g* for 30 min, and stored at -20°C until extracted from cell pellets. For chili powder, metagenomic samples were first vigorously mixed, passaged through Miracloth filter membrane (EMD Millipore, Billerica, MA, United States), and then sedimented at 200 × *g* for 3 min, to remove chili powder from bacterial cells, prior to harvesting the cell pellets by centrifugation, as described above.

### Sequencing of Microbiome Samples from Food

Genomic DNA from cilantro microbiome samples was extracted and 16S rRNA amplicon libraries were produced and sequenced as described in Daquigan et al. (2016). Genomic DNA from the ice cream microbiome samples was extracted and 16S rRNA amplicon libraries were produced and sequenced as described in Ottesen et al. (2016). Genomic DNA from chili powder samples was extracted using the DNeasy Blood and Tissue kit (Qiagen, Germantown, MD, United States). 16S rRNA community profile sequencing of chili powder microbiome samples was performed as described by Ottesen et al. (2016). Raw sequencing reads were subjected to preprocessing, as described in Section “Diagnostic True Positive Rate for *S. enterica* Detection,” and submitted for high-resolution taxonomic identification.

### Analysis of Bacterial Blood Stream Infection 16S rRNA Dataset

16S rRNA sequence data generated on the Illumina MiSeq platform utilizing paired-end sequencing were processed as follows: Raw overlapping paired-end reads were merged into consensus fragments by FLASH (Magoc and Salzberg, 2011) requiring a minimum 20 bp overlap with 5% maximum mismatch density, and subsequently filtered for quality (targeting error rates < 1%) and length (minimum 200 bp) using Trimmomatic (Bolger et al., 2014) and QIIME (Caporaso et al., 2010a; Kuczynski et al., 2011b). Spurious hits to the PhiX control genome were identified using BLASTN and removed. Sequences were then trimmed of their associated primers, evaluated for chimeras with UCLUST (*de novo* mode) (Edgar et al., 2011), and screened for human-associated contaminant using Bowtie2 (Langmead and Salzberg, 2012) searches of NCBI Homo sapiens Annotation Release 106. Mitochondrial contaminants were detected and filtered using the RDP classifier (Wang et al., 2007) with a confidence threshold of 50%, and passing high-quality 16S sequences were assigned to a high-resolution taxonomic lineage using the Insight method. Species level assignments that were present with at least 10 reads in at least two negative control samples were designated as contaminants and removed from downstream analysis. Patients with fewer than 500 final sequences per duplicate MiSeq run were removed from analysis.

## Results

### Accuracy for *S. enterica* Identification

**FIGURE 1:**
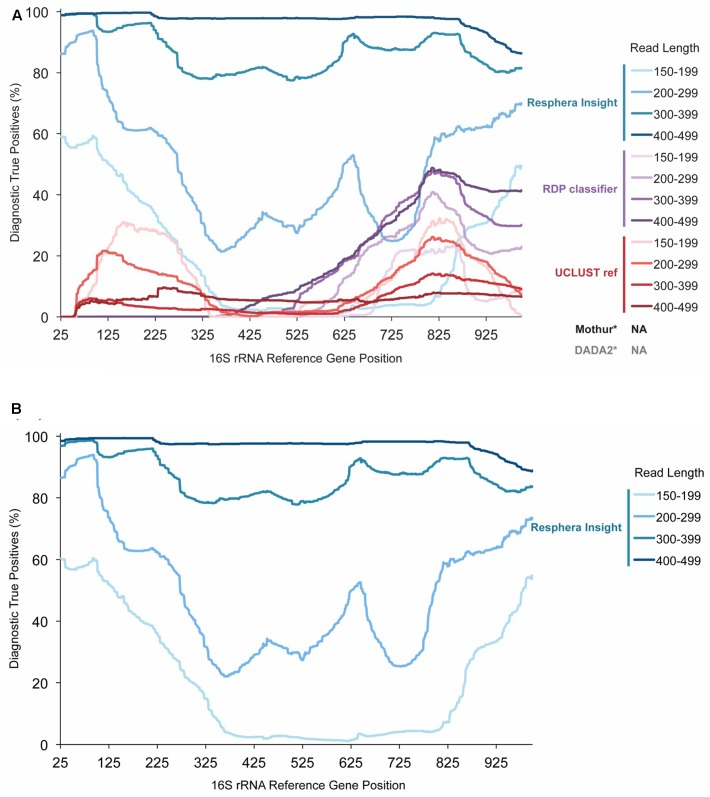
*Salmonella enterica* Diagnostic True Positive Rate of Resphera Insight and 2 other taxonomy assignment tools. **(A)**. Performance of the Insight method and other tools on 512 isolates of *S. enterica*. **(B)**. Expanded validation of Resphera Insight on 12,090 isolates of *S. enterica. Y*-axis shows the Diagnostic True Positive Rate (DTP), i.e., the percentage of sequences assigned unambiguously to *S. enterica*. Lines display the average DTP for all reads covering each gene position for a given read length range. DTP rates are improved for Resphera Insight with longer sequences that cover the first 250 bp of the 16S rRNA gene. ^∗^The default versions of Mothur and DADA2 do not generate species level assignments.

We assessed how performance of three 16S rRNA signature identification algorithms, Insight, RDP, and UCLUST, changed according to changing sequencing read length and 16S rRNA gene position. Overall, across the V1–V3 region (16S rRNA gene positions 27–534), Resphera Insight achieved DTP rates up to 99.7%, with improved accuracy correlated with increased read length (**Figure 1A**). For sequences less than 300 bp, sensitivity was significantly reduced. Among positions in the same region, RDP and UCLUST were unable to achieve a DTP above 11% for reads greater than 300 bp. Interestingly, the UCLUST method had improved sensitivity with shorter read lengths, though performance remained generally poor with a maximum DTP of 31.1% (**Figure 1A**). It should be noted that the DTP rates for all methods evaluated tended to dip in value across the 16S rRNA sequence that corresponds to the 3′ end of the V3 region, and this reduction in diagnostic capability was more pronounced for shorter read lengths (**Figure 1A**).

As the Insight method had the highest DTP rates, an expanded evaluation was then performed on all *S. enterica* isolates annotated in GenomeTrakr available at the time (*n* = 12,090, Supplementary Table S2). In this larger evaluation, we observed a very consistent detection profile, nearly identical to that seen for the 512 random subset dataset (**Figure 1A**), including high accuracy within the first 250 bp of the 16S rRNA gene for sequences that are at least 300 bp in length (**Figure 1B**).

### False Positive Rates for *S. enterica* Detection

**FIGURE 2:**
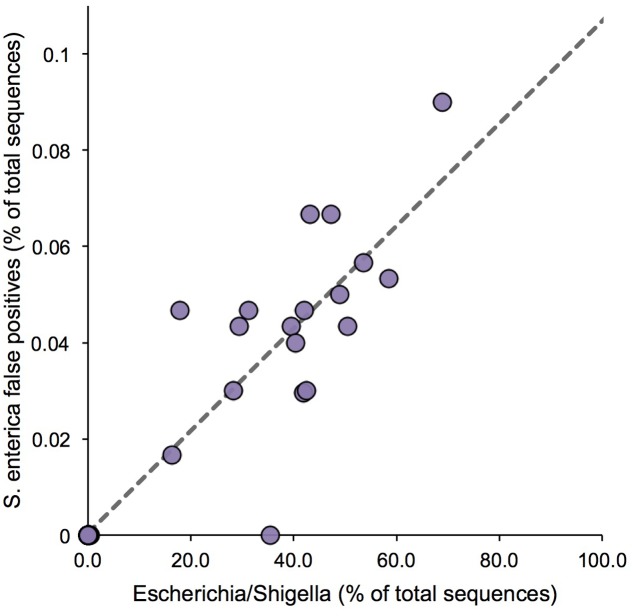
False positive assignments to *Salmonella* for Resphera Insight. Sequencing of spiked samples of raw cheese milk demonstrates a positive correlation between *Escherichia*/*Shigella* abundance and false positives assignments to *S. enterica*. Linear regression (dashed line) estimates a maximum predicted *S. enterica* false positive rate of 0.11%.

In addition to assessing the accuracy to detect *Salmonella*, we also sought to define the Type I error, or false positive rate, associated with detection of *S. enterica*; i.e., cases in which the algorithm incorrectly assigned a sequence to *S. enterica.* As *S. enterica* is closely related to *E. coli*, a false positive rate assessment was performed, utilizing 36 raw milk cheese samples spiked and selectively enriched with variable levels of *E. coli*, to establish how frequently an *E. coli* fragment would be incorrectly assigned to the target organism, *Salmonella*. Relative abundance estimates of *Escherichia/Shigella* ranged from 0.02 to 68.8% across the sample set, and we found a significant positive association between *Escherichia/Shigella* abundance and false positive assignments to *S. enterica* (*R*^2^ = 0.83, *F*-test *P* < 1e-4; **Figure 2**). Simply stated, the more *Escherichia/Shigella* 16S rRNA sequences contained in the microbiome sample, in terms of relative abundance, the more likely it was to produce a *Salmonella* false positive. However, using linear regression, the maximum predicted false positive rate was only 0.11%, given a completely homogenous community comprised entirely of *E. coli* (**Figure 2**).

Moreover, *in silico* simulations in which sequencing reads are composed entirely of *Enterobacteriaceae* 16S rRNA gene sequences and no background flora, utilizing artificial reads from two amplicon regions, V1 – V3 and V3 – V4, of the 16S rRNA gene from pure isolates of 15 different *Enterobacteriaceae* members, indicate that false positive rates for *Salmonella* detection approach 0.2%, when closely related members, such as *Citrobacter* and *Enterobacter* spp., are dominant members in a microbial community (**Table 1**). Interestingly, false positive rates were only observed for those *Enterobacteriaceae* species that are phylogenetically close to *Salmonella*, with, on average, slightly higher false positive rates observed for the V3 – V4 region (**Table 1**).

**Table 1:** *Salmonella enterica* false positive rates for V1–V3 and V3–V4 16S rRNA amplicon regions.

	*S. enterica* % false positives	
	
Species	V1–V3	V3–V4
*Enterobacter cloacae*	0.030	0.200
*Leclercia adecarboxylata*	0.000	0.090
*Enterobacter aerogenes*	0.000	0.061
*Citrobacter amalonaticus*	0.040	0.040
*Enterobacter cancerogenus*	0.010	0.022
*Klebsiella pneumoniae*	0.000	0.021
*Enterobacter hormaechei*	0.040	0.020
*Escherichia coli*	0.020	0.010
*Citrobacter freundii*	0.000	0.010
*Proteus mirabilis*	0.000	0.000
*Serratia marcescens*	0.000	0.000
*Yersinia enterocolitica*	0.000	0.000
*Klebsiella oxytoca*	0.000	0.000
*Proteus mirabilis*	0.000	0.000
*Serratia marcescens*	0.000	0.000


### Identification of *S. enterica* in Food Samples

To demonstrate and evaluate the utility of Resphera Insight to detect *S. enterica* and characterize overall microbial community composition, we performed 16S rRNA community profiling on microbial enrichment samples of (i) ice cream, (ii) cilantro, and (iii) chili powder. All three datasets included samples that were spiked with *S. enterica* and samples that were unspiked. Aliquots for 16S rRNA community profiling and metagenomics were taken at procedural time points in the enrichment protocols, namely 0 h (unenriched, for all three samples), 24 h (following non-selective enrichment for cilantro and chili powder and midpoint of non-selective enrichment for ice cream), and 48 h (following non-selective enrichment for ice cream samples and following selective RV and TT enrichment for cilantro samples). Due to the low inoculation level used in the cilantro and ice cream experiments, *Salmonella* could not be detected in any of the 0-h (un-enriched) samples, at the sequencing depth utilized (data not shown). Accordingly, 0-h chili powder samples were also excluded from the analyses.

**FIGURE 3:**
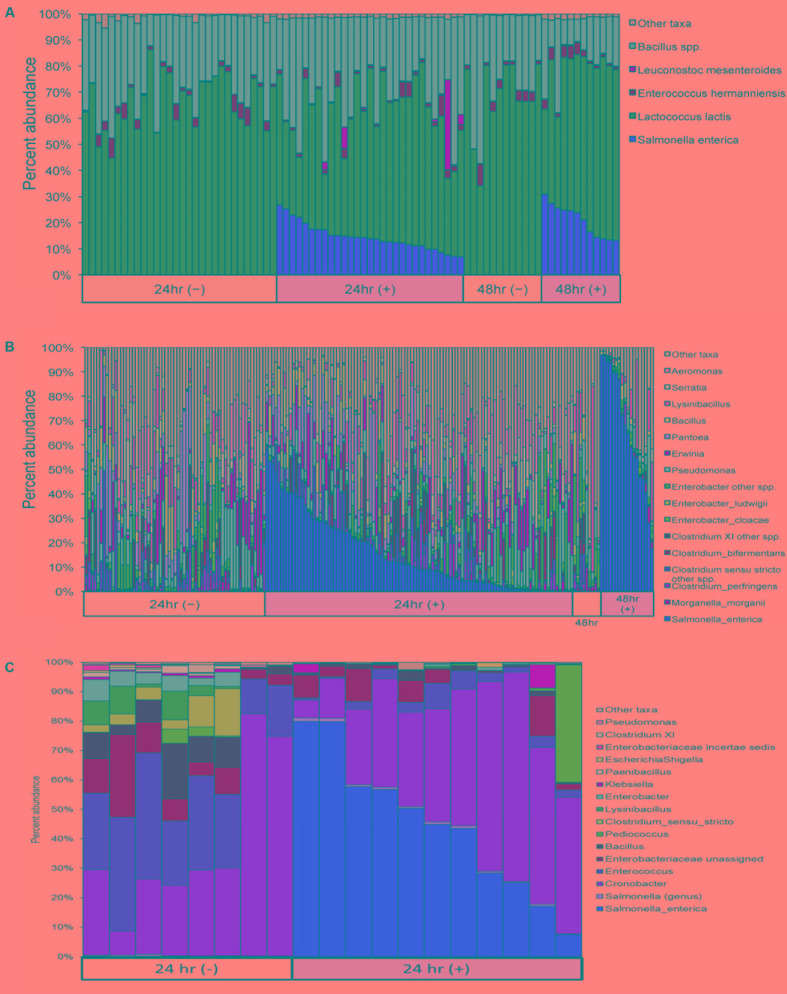
Microbiome profiles for **(A)** ice cream and **(B)** cilantro samples after 24 and 48 h enrichments, and **(C)** chili powder samples after 24 h enrichments, sorted by *Salmonella* culture status (±). We detect *S. enterica* with at least 0.1% abundance in 98% of *Salmonella* culture-positive samples. Ice cream samples are dominated by *Lactococcus lactis*, while cilantro- and chili powder-associated communities harbor increased overall diversity including members of *Clostridia* and *Enterobacter*.

*Salmonella enterica* relative abundances in ice cream enrichments ranged as follows: 0.000 to 0.004% (24 h, culture-negative); 0.000 to 0.008% (48 h, culture-negative); 7.018 to 26.742% (24 h, culture-positive); and 13.272 to 30.842% (48 h, culture-positive) (**Figure 3A** and Supplementary Table S3). Ice cream samples showed low overall bacterial diversity and low initial bacterial load, approximately 200 CFUs per gram. The dominant organisms, during and following enrichment, were *Lactococcus lactis*, *Bacillus* spp., *Enterococcus hermanniensis*, and if spiked, *S. enterica.*

Among cilantro samples, *S. enterica* relative abundances ranged as follows: 0.000 to 0.140% (24 h, culture-negative); 0.000 to 0.500% (48 h, culture-negative); 0.024 to 58.568% (24 h, positive); and 2.757 to 96.936% (48 h, positive) (**Figure 3B** and Supplementary Table S3). The un-enriched microbiomes from cilantro samples had considerably higher bacterial loads, 2 × 10^7^ CFU per gram on average, and higher diversity than ice cream (**Figures 3A,B**). In addition to *Salmonella*, the non-selective enrichment also enriched several other *Enterobacteriaceae* species, including *Enterobacter cloacae*, *E. ludwigii*, *Morganella morganii, Pantoea* spp., *Erwinia* spp. and *Serratia* spp. The relative abundance of several other taxa also increased from un-enriched to enriched time point samples, including *Aeromonas*, *Lysinibacillus*, and several *Clostridium* species, such as *Clostridium perfringens* and *C. bifermentans. Pseudomonas* was commonly found to be the dominant member of un-enriched cilantro samples (data not shown), and it persisted in the enriched samples, although its relative abundance decreased (**Figure 3B**).

For chili powder, *S. enterica* relative abundances ranged as follows: 0.010 to 0.226% in 24 h culture-negative samples and 7.392 to 79.920% in 24 h culture-positive samples (**Figure 3C** and Supplementary Table S3). The un-enriched microbiomes from chili powder samples were similar in terms of diversity to those of cilantro, but less abundant, 5 × 10^5^ CFU per gram on average. Similar to results from cilantro samples, the non-selective enrichment step not only enriched for *S. enterica*, but also enriched several other *Enterobacteriaceae* species, in this case, *Cronobacter*, *Enterobacter, Klebsiella*, and *Escherichia*/*Shigella.* Many of these taxa were dominant members of the un-enriched microbiome, and they persisted through the non-selective enrichment step (**Figure 3C**). The relative abundance of *Enterococcus* and *Pediococcus* also significantly increased from un-enriched to enriched time point samples (**Figure 3C**).

Due to the intrinsic “noise” of 16S rRNA-based community profiling, investigators are forced to choose a minimum abundance threshold, defined as the percentage of reads assigned to *S. enterica* in a sample, to determine the outcome of a diagnostic assignment (presence/absence). *Salmonella* detection was evaluated using inoculated cilantro, chili powder and ice cream enrichments at four minimum abundance thresholds (0.01, 0.10, 0.20, and 0.25%) and compared to culture results. Sensitivity and specificity was 100% in all ice cream samples regardless of the *Salmonella* threshold utilized for analysis (**Tables 2**, **3**). Using a 0.10% relative abundance threshold, the algorithm gave a 98% correct classification rate, or sensitivity, across all three commodities for *Salmonella*-positive (*n* = 174) samples (**Table 2**). A 96% true negative rate was observed for *Salmonella*-negative (*n* = 120) samples using this threshold (**Table 3**), indicating the tool was both specific and sensitive for detecting *Salmonella* from food samples.

**Table 2:** *Salmonella enterica* sensitivity rates by limit of detection threshold (0.01, 0.10, 0.20, and 0.25%).

Sample type	Culture result	Enrichment time	Sensitivity rate by threshold			
			
			0.01%	0.10%	0.20%	0.25%
Cilantro	Positive	24 h (*n* = 104)	100%	97%	93%	91%
		48 h (*n* = 18)	100%	100%	100%	100%
Ice cream	Positive	24 h (*n* = 29)	100%	100%	100%	100%
		48 h (*n* = 12)	100%	100%	100%	100%
Chili powder	Positive	24 h (*n* = 11)	100%	100%	100%	100%
		Total (*n* = 174)	100%	98%	96%	95%


**Table 3:** *Salmonella enterica* specificity rates by limit of detection threshold (0.01, 0.10, 0.20, and 0.25%).

			Specificity rate by threshold			
			
			0.01%	0.10%	0.20%	0.25%
Cilantro	Negative	24 h (*n* = 61)	38%	98%	100%	100%
		48 h (*n* = 9)	33%	89%	89%	89%
Ice cream	Negative	24 h (*n* = 30)	100%	100%	100%	100%
		48 h (*n* = 12)	100%	100%	100%	100%
Chili powder	Negative	24 h (*n* = 8)	12.5%	62.5%	87.5%	100%
		Total (*n* = 120)	58%	96%	98%	99%


We observed low specificity to detect *S. enterica* in cilantro and chili powder samples when using the lower minimum abundance thresholds. For example, lowering this *Salmonella* threshold from 0.10 to 0.01% increased the sensitivity to 100% for all *Salmonella-*positive cilantro enrichments, but lowered the specificity to 38 and 33% in *Salmonella*-negative 24 and 48 h enrichments, respectively (**Table 3**). Conversely, compared to the 0.10% threshold, a 0.20% threshold increased the specificity to 100%, but decreased the sensitivity to 93% for 24 h cilantro enrichments. Similarly, increasing the threshold to 0.25% increased the specificity to 100% but also decreased the sensitivity to 91% (**Table 3**).

In *Salmonella-*negative 48-h enrichments from cilantro samples, the 89% specificity was consistent for the 0.10, 0.20, and 0.25% thresholds. One *Salmonella*-negative cilantro sample (48 h, RV enrichment) resulted in an unusually high *S. enterica* mis-assignment rate of 0.5%; however, we did not observe a similar effect in its treatment pair (48 h TT broth). Further inspection of these pairs revealed that the false positive RV sample harbored a higher abundance of *Enterobacter* specie*s* (e.g., *E. ludwigii, E. cloacae*) compared to its TT pair (36.5 vs. 14.8%). This excess diversity of closely related species may have resulted in low frequency chimera formation during PCR not easily identified during preprocessing, which may be assigned to *Salmonella*.

A similar pattern was observed for the chili powder samples, even though the number of *Salmonella*-positive and -negative samples were low. This commodity also had a high number of other *Enterobacteriaceae* members, which may have resulted in chimeras not removed from the dataset during preprocessing, and contributed to a higher rate of misidentifications as *S. enterica*. This situation would also likely lead to a slight over-representation of the target organism, *S. enterica* in positive samples, as well.

### Application to Diagnosis of Bacterial Bloodstream Infection

In addition to detection of *S. enterica* in food-associated microbial communities, we sought to apply this approach to a recent study of children with severe febrile illness, reported by Decuypere et al. (2016). This study included confirmed culture positive cases of *S. enterica* bloodstream infections in a subset of children. We obtained and reprocessed the original 16S rRNA amplicon sequences from two replicate MiSeq runs (Run1 and Run2), requiring that passing samples have at least 500 high-quality 16S rRNA sequences in both runs after removal of bacterial contaminants identified in negative controls (see Materials and Methods). This resulted in a final dataset of 26 pediatric patients, three of whom were blood culture positive for *S. enterica* (two for *S. enterica* serovar Enteritidis and one for *S. enterica* serovar Typhimurium).

**FIGURE 4:**
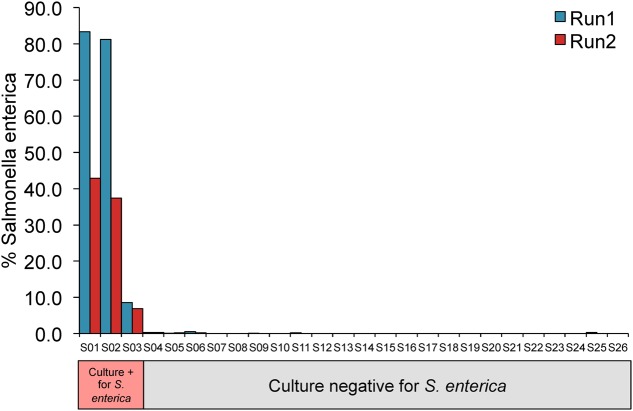
16S rRNA profiling of blood samples are consistent with *Salmonella* culture results. *Y*-axis displays percentage of total sequences assigned to *S. enterica*.

All three patients positive for *S. enterica* were correctly identified in both MiSeq runs, all with *S. enterica* abundances greater than 6% (**Figure 4** and Supplementary Table S4). *S. enterica* sequences were also detected in three additional patients that were concordant across both replicate runs, however, each of these samples harbored less than 0.5% of the organism. *Salmonella* sequences were identified in the original study at a low level in the majority of samples, suggesting a contaminant background of *Salmonella* that would explain these culture negative findings.

## Discussion

Overall, these data provide a comprehensive assessment of the ability to detect *S. enterica* with high specificity and sensitivity using Resphera Insight high-resolution microbiome profiling. When compared against two commonly used algorithms for assigning taxonomy within QIIME (Caporaso et al., 2010b), the tool performed significantly better in terms of Diagnostic True Positive rate, which translates into a higher proportion of true *S. enterica* sequences being assigned correctly to this species. Unclassified assignments with existing tools continue to be an area of concern even though the amount of 16S rRNA sequences grows exponentially in this WGS era, highlighting the need for tools such as this high-resolution microbiome profiling algorithm and database to help accurately classify, or assign taxonomy, to large microbiome datasets.

We observed a similarly high specificity for *S. enterica*, or true negative rate. Using *in silico* simulations of other *Enterobacteriaceae* and raw milk cheese spiked with *E. coli*, we demonstrated that the diagnostic false positive rate for *S. enterica* was never above 0.11%. This high specificity was confirmed with low-diversity ice cream samples that were culture-negative for *Salmonella*. However, when microbiome samples, such as cilantro and chili powder, contained a more complex microbiome, including several closely related enteric species, the specificity of the tool fell dramatically at the lowest minimum abundance detection threshold tested. In terms of bacterial biomass, it is notable that the 24-h *Salmonella* positive cilantro samples had levels of *Salmonella* ranging from 7.44 to 8.74 log MPN -g cilantro with corresponding background bacterial concentrations of 9 to 10 log CFU -g (Daquigan et al., 2016). In comparison, the 24-h ice cream samples with less diverse microbiomes, harbored bacterial concentrations of approximately 6.43 log MPN -g and 10.54 log CFU -g for *Salmonella* and background microorganisms, respectively. This further demonstrates the higher impact of microbiome diversity on specificity even when concentrations of cultured background microorganisms and *Salmonella* were similar. We suspect that the presence of closely related species impacts the specificity of the algorithm through more false positives to *Salmonella* due to “noisy” low frequency sequences that are mis-identified and a higher formation of chimeras during PCR that may not be adequately filtered by preprocessing. However, it should be mentioned that these challenges are a characteristic of the dataset and would apply to all analytical tools used.

The decrease in specificity could be resolved, by moving the minimum abundance threshold higher. However, this resulted in a loss of sensitivity with samples in which the relative abundance was below the minimum abundance threshold. Fortunately, 16S rRNA metagenomics gives a complete picture of the microbial community sequenced, so that the detection threshold can be adjusted accordingly based on the microbial composition. Based on this study, a 0.20% relative abundance threshold for *S. enterica* was established with an overall sensitivity of 96% and specificity of 98%. When this 0.20% threshold was used to detect *Salmonella* from bloodstream samples, all three culture-positive patients were identified, supporting the notion that accurate identification of *Salmonella* can be performed in clinically relevant scenarios, as well as food safety surveillance.

Given the increasing level of interest in microbiome analysis by food safety stakeholders, this study suggests that microbiome sequencing diagnostic tools to identify pathogens need to be carefully designed and implemented, with rigorous evaluations to determine sensitivity and specificity, as demonstrated here. In addition to downstream analytical tool choice, all other aspects of this workflow, such as sample collection and DNA extraction, 16S rRNA amplification and sequencing strategy, must also be evaluated. For example, as demonstrated in this study with *Salmonella* and, in a previous study of *Listeria*, targeting the V1 to V3 region of the 16S rRNA gene improves sensitivity and specificity of detection of these pathogens (Ottesen et al., 2016). However, this may not be the case with all organisms; for example, *Bacillus anthracis* can be distinguished from members of the *B. cereus* group by targeting the V6 region (Chakravorty et al., 2007).

Due to intrinsic noise in 16S rRNA community profiling, even tools such as Resphera Insight, which demonstrated high specificity and sensitivity, show limitations and challenges in their usage. As shown in this study, detection of *S. enterica* will depend not only on its absolute abundance, but also its relative abundance compared to the background microbial population and the complexity of that population. False positive assignments, though infrequent, do occur suggesting that identification of *S. enterica* below 0.20% abundance may be challenging for some samples in which other closely related *Enterobacteriaceae* members dominate a community. However, for microbiological research, the ability to characterize the microbial population and its changes can be beneficial for studying how experimental parameters and sample sources, such as different food matrices, affect target recovery.

## Author Contributions

CG, ND, and JW drafted and wrote the manuscript. CG, ND, TL, AO, and KJ designed and conducted the experiments. CG, ND, KJ, and JW analyzed and interpreted the data. JW performed the bioinformatics analyses. All authors read and approved the final manuscript.

## Disclaimer

Use of trade names and commercial sources is for identification purposes only and does not imply endorsement by FDA or the United States Department of Health and Human Services.

## Conflict of Interest Statement

JW is founder of Resphera Biosciences and has an equity position in the company. The other authors declare that the research was conducted in the absence of any commercial or financial relationships that could be construed as a potential conflict of interest.

## Footnotes

1 www.respherabio.com
